# Food and nutrition labelling in Thailand: a long march from subsistence producers to international traders

**DOI:** 10.1016/j.foodpol.2015.07.011

**Published:** 2015-10

**Authors:** Wimalin Rimpeekool, Sam-ang Seubsman, Cathy Banwell, Martyn Kirk, Vasoontara Yiengprugsawan, Adrian Sleigh

**Affiliations:** a National Centre of Epidemiology and Population Health, Research School of Population Health, Australian National University, 62 Mills Road, Canberra, ACT 2601, Australia; b School of Human Ecology, Sukhothai Thammathirat Open University, Chaengwattana Rd, Muang Thong Thani, Bangpood, Nonthaburi 11120, Thailand

**Keywords:** Food label, Nutrition label, World Trade Organization, History, Thailand

## Abstract

This paper reviews the evolution of Thai food and nutrition label policies and Thailand’s international role relating to food product safety and standards. The historical record has been interpreted to identify future trends and challenges related to food labelling. These challenges are arising in Thailand and many similar emerging economies.

Thailand has a good reputation in world food markets and is now becoming a global leader in food production and export. It has become deeply involved with regulations and standards applied by World Trade Organization and Codex Alimentarius while serving its own population with a safe and secure food supply. For consumers considering Thai food products, food labels can provide useful nutrition information and help build trust.

Thais began a century ago with policies and laws to enhance food safety and to protect Thai consumers. During the lengthy journey from national to global standards Thai food labels have evolved and now contribute to international food labelling policies. This contribution comes from the perspective of a leading middle income south-east Asian food producer now trading with high income countries around the world. The story of that journey – a case study for many other countries in a similar situation – has not previously been told.

This article provides information for policy makers dealing with food labelling, embedding trends and tensions for one middle income food exporter in a long history. Information captured here should be helpful for other middle income countries, especially those with limited records. This strategic knowledge will enable better decisions for future policies.

## Highlights

• Thai food labels show transition from closed agriculture to global agribusiness
• Thai food quality follows investment in food science and technology after WW2
• Thai food labels address transition from hunger to plenty and international rules
• It is difficult to harmonize nutrition labels among the countries of SE Asia
• Domestic protection versus global food trade creates tension and new rules

## Introduction

Labels have long played a part in promoting food products all around the world. Over the last century, food labels also have become increasingly involved in consumer protection by including information regarding both safety (Marks, 1984) and nutritional content (Taylor and Wilkening, 2008a,b). As countries develop their food systems, food labelling plays an important role because good practices and improved food safety are the usual consequence. A transition to food quality and safety is also proceeding in countries undergoing rapid development from traditional subsistence to modern middle income status. Now many countries around the world face difficulties balancing national nutrition, consumer protection, and international trading agreements. A good example of such a transition is Thailand.

In Thailand, food labels first were used to protect consumers from adulterated imported foods. To ensure food safety and quality, the Thai government, through its Ministry of Public Health (MOPH), assumed responsibility for food labels and related policy amendments. Now food labelling reflects and enhances the trustworthy image of Thai food exports.

More than 100 years ago, the Thai government issued its first regulation for food. It prohibited the sale of contaminated or adulterated food from 1908. The first food labelling requirement came much later in 1941. The evolving Thai system of food labels has since gradually harmonized with international developments codified by key United Nations (UN) agencies including the Food and Agricultural Organization (FAO), the World Health Organization (WHO), the Codex Alimentarius, and the World Trade Organization (WTO). The developing food industry, consumer movement, international trade, and health transition are all involved in changes of food label regulations. Thailand now has a good reputation for food production and food labelling has contributed to this reputation.

This paper assembles and interprets historical and contemporary data on food and nutrition labelling policy in Thailand. Such historical analysis has never been reported. By understanding the historical development of food labelling, and concomitant social changes, policy makers will be better positioned to anticipate and shape the future. Government regulators should understand the history of labelling in their own jurisdictions. This knowledge will facilitate development of new labelling policies that respond to prevailing nutrition problems as well as helping design the food labels of the future. As well this information should inform debate on fair trade and consumer protection of other countries in a similar situation to Thailand. Obviously, the information we present will be most relevant to the Southeast Asia region but will also help in many other parts of the world.

## Methods

National and international databases and key Thai document collections were searched for information on food labelling. The search ended in May 2015. Data were collected from the Royal Thai Government Gazette e-database, the largest collection of Thai laws, registrations, and notifications. This database was searched in Thai using five keywords with results as follows: 7 documents for ‘food label’, 4 documents for ‘nutrition label’, 512 documents for ‘label’, 330 documents for ‘food MOPH notifications’ and 44 documents for ‘packaged food’. Each of these 897 documents was examined and those with substantive information relevant to food labelling, or to related aspects of food regulation or the food industry, were placed in an annotated computer file (*n* = 137).

The search for information also extended to published articles and monographs. First the Thai Food and Drug Administration (Thai FDA) e-library was searched in Thai for the term ‘food label’ and ‘nutrition label’, yielding 30 relevant articles. Then the international ScienceDirect, Medline, and Scopus databases were searched in English looking for publications with the term ‘Thai or Siam’ in all fields, and the term ‘food or nutrition’ and ‘label’ in the title field. All terms were entered with a wildcard to allow for truncation, yielding 11 unique articles. In 3 of these 11 articles the reference to Thailand involved no more than one or two sentences and the other eight were not relevant. Finally, to complete a thorough review of information bearing on food labelling, 19 rare old documents in Thai were found in the Kasetsart University Knowledge repository e-database; as well in the Thai government Department of Science Service digital archive the DSS bulletin was scanned (100 documents) and three issues contained relevant information.

The screening described above resulted in a total of 189 (137 + 30 + 19 + 3) documents available for the next stage of analysis. Each was then read fully and some documents were found to duplicate information or contained excessive detail. Eventually 39 of these documents or articles were actually used in this report along with other references that were identified through cross-citations. All these are listed with the references.

All the significant laws and documents found relevant to Thai food labels were dated between 1908 and 2014. For this report, the information gathered on food and nutrition labelling covering the last century is organized into six sections. These include the start of Thai food industry and its regulation, early experiences of labelling, modern food labelling, nutrition labelling, Thai nutrition label challenges, and international tensions.

## Results

### Beginning of food industry and its regulation in Thailand (1906–1944)

The first phase of food regulation in the Kingdom of Siam (previous name of Thailand) responded to imported low quality or adulterated foods. Such foods were widespread at the start of the 20th century. A report about spoiled tinned food had appeared in the Journal of the Siam Society as early as 1906. It noted that food producers were not required to stamp the canning date on each tin. Old stocks that should have been destroyed were sold to the small traders (Highet et al., 1906). In 1908, new regulations in many fields were introduced as part of a modern penal code for the Kingdom of Siam (Thai FDA, 2009). The new law does not mention food labels or canning dates but use of false brands or names on products was specified as infractions of the code (Royal Thai Government Gazette, 1908).

In the late 1910s, skimmed milk was considered to be a food lacking in nutritive value because butterfat, carrier of vitamin A, was removed (Howard, 2013). Many Thai physicians agreed that skimmed milk could not give infants enough nutrients and might cause sickness. The Skimmed Milk Act in 1927 controlled imported milk (Royal Thai Government Gazette, 1927). At this point, the Thai government developed a food quality analysis unit to measure the mineral composition and quality of milk. It was located in the existing Government Laboratory in Bangkok (*Salayaekthatu*) that was responsible for geological analyses (Ministry of Industry, 1953). The Skimmed Milk Act 1927 was the first attempt to protect consumers from fake foods. It led to development of laboratory expertise and food science needed to investigate the composition of foods and validate the labels.

After World War 2 Thailand became an early member of the UN. It joined the FAO in 1947 and began to industrialize. For the food industry a Department of Science (DOS) evolved incorporating the Government Laboratory. Opportunities grew for international knowledge exchange. For example, FAO sent a specialist to Thailand to work on food and nutrition with the DOS and the MOPH in 1955. A UN scholarship was given to a government scientist to visit Australia to study food processing in 1957 and another Thai scientist was sent to study food canning and preservation in Denmark in 1964 (Bhumiratana, 1966a,b; Ministry of Industry, 1955a,b). Thai food industries responded well and modern food science and technology appeared quickly in the 20 years after World War 2.

Food preservation industries became prominent in Thailand during the 1950s. At first, the Thai government developed a pilot food factory, the Preserved Foods Organization (PFO) established in 1955, managed by the Ministry of Defence. The PFO aimed to produce instant or ready-to-eat foods that could be used during a civil or military emergency (Royal Thai Government Gazette, 1955). As well knowledge about combat rations was obtained from US Armed Forces. The first prototypes produced by PFO were canned rice and food dishes exhibited at the first Thai Trade Fair (1962). Three years later, the PFO developed dehydrated combat rations (instant rice and dried banana in tin foil) for military use (Bhumiratana, 1966a). As a knowledge hub, PFO was an important influence on food laws in later decades.

The Thai canned foods industry arose in the 1950s and was the first food industry component to receive strong government financial and technical support. The aim was to stimulate consumer demand and to reduce food imports when food shortages appeared after World War 2 (Royal Thai Government Gazette, 1956). The first generation of Thai canned foods were produced by small enterprises with simple production practices (Ministry of Industry, 1975), such as canned pickled mustard greens in 1950 (The Peace Canning (1985) Co. Ltd., 2010) Much later, large canned food companies introduced modern production systems. This led to internationally famous exports including Thai pineapples in 1967 (Siam Agro-Food Industry Public Company Limited, 2013). This expertise with canned foods and tropical fruits continues up to the present day.

### Early Thai food labelling

The Food Quality Control Act 1941 was the first comprehensive Thai food law and it defined the word “label” to be any statement or picture or imprinting on food, box, package, or container. Food products with labels that misled consumers about quality, quantity, or specific characteristics were classified as fake food. A Thai food label with the name and address of the place of business was required when food was mixed with a number of ingredients or was sold with a special name (Royal Thai Government Gazette, 1941). The law protected consumers from fake foods but there was no overall guidance for labelling details.

One year later, in 1942, foods which included colourants were the first group specified to require food labelling. Colourant-added-products (1) shall have a Thai label and if the label also displays in a second language, it shall not have a different meaning, (2) specified colourants shall be declared. As well, milk had to declare the ‘type of food’ and ‘place of production’ for many types of products such as condensed milk, milk powder, and colostrum (Royal Thai Government Gazette, 1942a,b). Non-declaration could lead to court proceedings. However, government capacity was limited and the initial focus was limited to colourants and milk products (Deputy Director General of Department of Science, 1942).

The Food Quality Control Act 1941 consolidated in the years through to 1959 and new food controls soon appeared. Low quality canned food (both Thai and imported) had become widespread in Thailand after World War 2 and the existing laws could not make this food safe. As well, an increasing number of bad quality canned foods displayed fake labels. For example, canned rambutan products were labelled with a photo of lychees; or food producers tried to conceal their place of production by using English language labels to make consumers think it was an imported product. Some canned foods contained preservatives that were not declared. Canned foods became an important safety issue as reported in newspapers of the period (Bhumiratana, 1964, 1966b; The Thai Home Economics Association, 1965).

Many government groups met together for Thailand’s 11th PFO conference in 1962 to discuss the hot topic “How can we control [the quality of] processed food industries?” Staff from PFO, Ministry of Industry (MOI), and MOPH agreed that Thailand should amend food laws, especially for canned foods. They agreed that Thai food labels should name the food type and ingredients in food products (Bhumiratana, 1964). Two years later, in 1964, Notification No. 6 amended the Food Quality Control Act (1941, 1959) by requiring labels to present the truth. The type and name of food, place of production, quantity, and manufacturing date were required on the label (Royal Thai Government Gazette, 1963b).

At this point, international standards began to appear and Thailand often adopted them. The Director of Department of Science, Prof. Yos Bunnag, was on the committee drafting the Food Quality Control Act 1964 and he had participated in the Codex since the 1st Codex Alimentarius Commission (CAC) in 1963 (Pongsapitch, 2013; Royal Thai Government Gazette, 1963a). Thailand wanted to export food products, so it had to adopt the international standard of the Codex for safe food products, reducing trading barriers among countries (Thai FDA, 2000). This was the beginning of international food regulations and standards that Thailand adopted to use in later food laws.

The Food Quality Control Act 1959 had limited powers over food producers so a new Food Quality Control Act 1964 was promulgated (Bhumiratana, 1964). Food products with labels that deceive about quality, quantity, other special characteristics, place of production, or country-of-origin were classified as fake foods. The penalty for fake foods was imprisonment up to ten years or a fine not exceeding 20,000 Thai Baht (approximately US$600), or both. Accredited staff had authority to seize or destroy fake foods (Royal Thai Government Gazette, 1964a).

In 1964, the MOPH food label Notification No. 6 was the first label guided by the general provision of CAC. Food labels had to declare name, food registration number, net quantity and volume, and name and place of manufacture. The notification also covered declaration of any usage of preservatives or additives (including colourants, flavours, and antioxidants) with a specific statement and specified font size (Royal Thai Government Gazette, 1964b).

### Food labelling in modern Thailand (1979-)

The Consumer Protection Act 1979, and the Food Act 1979 which required product labelling without deception, were part of the consumer movement that swept the world at that time (Potipara, 2012). The Thai Food and Drug Administration (Thai FDA), created in 1974, was tasked to enforce these laws. The Food Act 1979 is the central law governing the food industry in Thailand today. It defines “food” as “edible items and those which sustain life”, including: (A) “substances eaten, drunk, sucked, or gotten into the body by mouth or other means … not including medicine, psychotropic substances or narcotics …”; (B) “substances for use as ingredients in production of food including additives, colouring, and flavouring”. The Act defines “label” as any symbols, pictures, printings or statements on food packages (Royal Thai Government Gazette, 1979a).

MOPH guidance in Thailand in 1979 indicated that food labels should display the name and type of food, food recipe registration number, name and location of manufacturer, manufacture date, quantity, and the ingredient list. Labels must mention any food preservatives, colourants, additives, and chemicals that were added. The label needs to be obvious and present the truth (Royal Thai Government Gazette, 1979b).

**Fig. 1:**
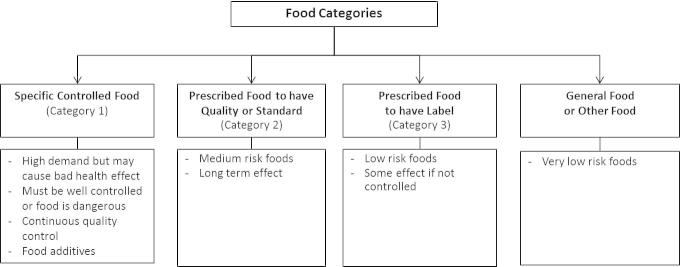
Food categories by Thai FDA regulations (Thai FDA, 1979).

**Fig. 2:**
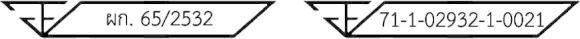
Thai FDA label before and after 2000.

Approved Thai FDA food labels for display on packages first began for “controlled” (Category 1) or “prescribed” (Category 2 and 3) foods in 1979 (Fig. 1) (Royal Thai Government Gazette, 1979b). Foods that must be labelled include products likely to cause adverse health effects if the quality is poor (Thai FDA, 1979). The Thai FDA “approval symbol” then came into use in 1985 showing consumers that the food labels had been approved by the MOPH (Fig. 2) (Royal Thai Government Gazette, 1985).

Further changes arose after Thailand became a WTO founding member in 1995 (World Trade Organization, 2014). The emphasis moved to post-marketing monitoring of food safety (Good Manufacturing Practice or GMP) and food quality (Thai FDA, 2000). In 2000, to support the free-trade system, “controlled” and “other foods prescribed by the Minister” became the only food groups that required label approval before sale (Royal Thai Government Gazette, 2000). The new Thai FDA approval symbol incorporated a thirteen digit food serial number. These numbers allow consumers to trace the food to its point of production (Fig. 2).

Some modifications were a result of cumulative small changes since 1979. Thus “net quantities” have been clarified and measurements are now metric (e.g. gram and millilitre). In 1981 “net quantity” evolved into two words – “net weight” (solids) and “net volume” (liquids). “Drained weight” quantifies chunky foods or foods packed in liquid, excluding foods which cannot be isolated from liquid. Since 1982, Thailand had required more comprehensive declarations with percentage of weight expressed in descending order of magnitude. Thailand was ahead of the Codex for a Quantitative Ingredients Declaration (QUID) (Royal Thai Government Gazette, 1979b, 1981, 1982; The International Association of Consumer Food Organizations, 2005).

Before the advent of the current 2014 rules regarding manufacturing date and expiration date, guidelines relating to these two dates had often been amended. Also, at first (1979), names and addresses of producers were simply expressed on the labels specifying the place of manufacture or the place of re-packaging. Finally, after many changes, in 2014 specific terminology was adopted including “Manufactured by”, “Repacked by”, “Headquarters”, or “imported by” (Royal Thai Government Gazette, 1979b, 2014).

Visibility and legibility of Thai food labels have evolved considerably. From 1979, the font size of certain key words such as “type of food” was fixed at not less than 5 mm, equivalent to Times New Roman 14 pt on a modern printer. Text colour should contrast to the background and font sizes should be appropriate for the label surface area. Labels should be placed on visible locations, and should be clear and easy to read. In 2014, a particular font size was indicated for specified text expressions. One millimetre is minimum font size required for smaller (<100 sq cm) package area (Royal Thai Government Gazette, 1979b, 2014).

One development yet to evolve in Thailand was the Principle Display Panel (PDP). In the USA, where PDPs were first developed, they were considered to be easily visible with a legible name, food registration number, place and address of manufacture, net weight, manufacturing date and expiration date. However, in Thailand PDP did not become the standard for labelling (Royal Thai Government Gazette, 1981, 1982, 2000).

Rules for food additives and preservatives continue to evolve. When the Food Act 1979 became law, manufacturers had to mention “utilizing preservative”, “colouring” or “flavouring”. In 1985, further division was required into “Natural flavour”, “Synthetic flavour”, and “Artificial flavour” – categories still used today. Since 2000, flavour enhancers and food sweeteners must be on the label. Since 2014, the food additives group has to include the corresponding number for the International Numbering System (INS) (Royal Thai Government Gazette, 1979b, 1985, 2000, 2014).

Another important change for labelling came in 2014 and is still in force (Notification No. 367). All pre-packaged foods (except fresh, kiosk and wholesale catering foods) now must have labels (Royal Thai Government Gazette, 2014). Allergen information labels are also now required for the first-time; this followed a recent study showing one-third of Thai commercial food products contained undeclared allergens greater than 10 ppm (Surojanametakul et al., 2012).

### Nutrition labelling in modern Thailand (1979-)

Thailand’s first nutrition label law was promulgated in 1998, nineteen years after the first food label law. Nutrition labelling laws were dependent on scientific discovery and this followed World War 2 and the collaborative work with foreign nutritionists (Kachondham et al., 1992). Many new scientific findings about diet and health led to Thai guidelines, and food and nutrient databases were created to improve Thai consumer knowledge.

Nutrition labels on foods were part of the national strategy to improve nutritional status of Thai people and followed after the first International Conference on Nutrition (ICN) was held in Rome in 1992 (Kongchuntuk and Intaraluk, 1999). At that conference, all countries agreed to make a World Declaration and Plan of Action for Nutrition. Nutrition labelling was part of a communication strategy to prevent diet-related non-communicable disease attributable in part to dietary and life style changes and urbanization. To harmonize labels among countries, each followed the international standards of the CAC (FAO/WHO, 1992). In the early 1990s, when Thailand was trying to grapple with serious problems of simultaneous under- and over-nutrition, the government adopted nutrition labels as one strategy for the 7th National Economic and Social Development Plan (1992–1996) (Kongchuntuk, 1996).

In 1990, the US Congress passed the Nutrition Labeling and Education Act (NLEA) (Taylor and Wilkening, 2008a), creating a problem for Thai food exporters because they had not previously had to display nutrition labels. In 1992, the Institute of Nutrition at Mahidol University (INMU) held a nutrition label workshop to help compliance with US food labelling laws. Beginning in 1993, chemical analyses of Thai foods involved cooperation between Thai food experts and food analysts (Pitsanupoom, 2001).

The format of the Thai nutrition label evolved in response to prevalent nutritional problems and was based on national priorities. A special project to develop Thai nutrition labels began in 1994 (Kongchuntuk, 1996) using the Codex guideline on nutrition labelling that had been available since 1985 (Codex Committee on Food Labelling, 2013). Thai FDA was in charge and cooperated with many national organizations including the INMU, as well as the MOPH Bureau of Nutrition, the MOI Office of the National Codex Alimentarius Committee, and the Federation of Thai Industries (Kongchuntuk and Intaraluk, 1999).

Recommended Daily Allowances for Healthy Thais (RDAs), created by MOPH in 1989, were not suitable for nutrition labelling because there are too many values dependent on age and sex (Royal Thai Government Gazette, 1998; Thai FDA, 1995). Accordingly, the Thai Recommended Daily Intake (Thai RDI) was established in 1995 to be a set of mean values for healthy Thai people above 6 years old. These values were created by choosing the highest nutrient value among Thai RDA, US Daily Values (DV), US Daily Reference Values (DRV), US Reference Daily Intakes (RDI), and Codex Nutrient Reference Values (NRV) (Kongchuntuk, 1996). Eventually, the Thai RDI specified 2000 kcal total energy, 65 g total fat, 300 g total carbohydrate, 2400 mg sodium and other nutrient values as standards (Thai FDA, 1995). For reference serving size, values came from consumer consumption surveys and from information provided by food producers (Royal Thai Government Gazette, 1998).

According to the Codex guideline, each country may require declaration of specific vitamins and minerals on nutrition labels (Codex Committee on Food Labelling, 2013). For Thailand, declarations of vitamin A, vitamin B1, vitamin B2, Calcium, and Iron are all mandatory under Thai nutrition label laws (Royal Thai Government Gazette, 1998). Those vitamins and minerals reflect the major nutrition problems noted in Thailand’s first Thai National Food and Nutrition Plan (NFNP) (1977–1981) (Kachondham et al., 1992). Vitamins and minerals required on Thai nutrition labels may differ from other countries.

**Fig. 3:**
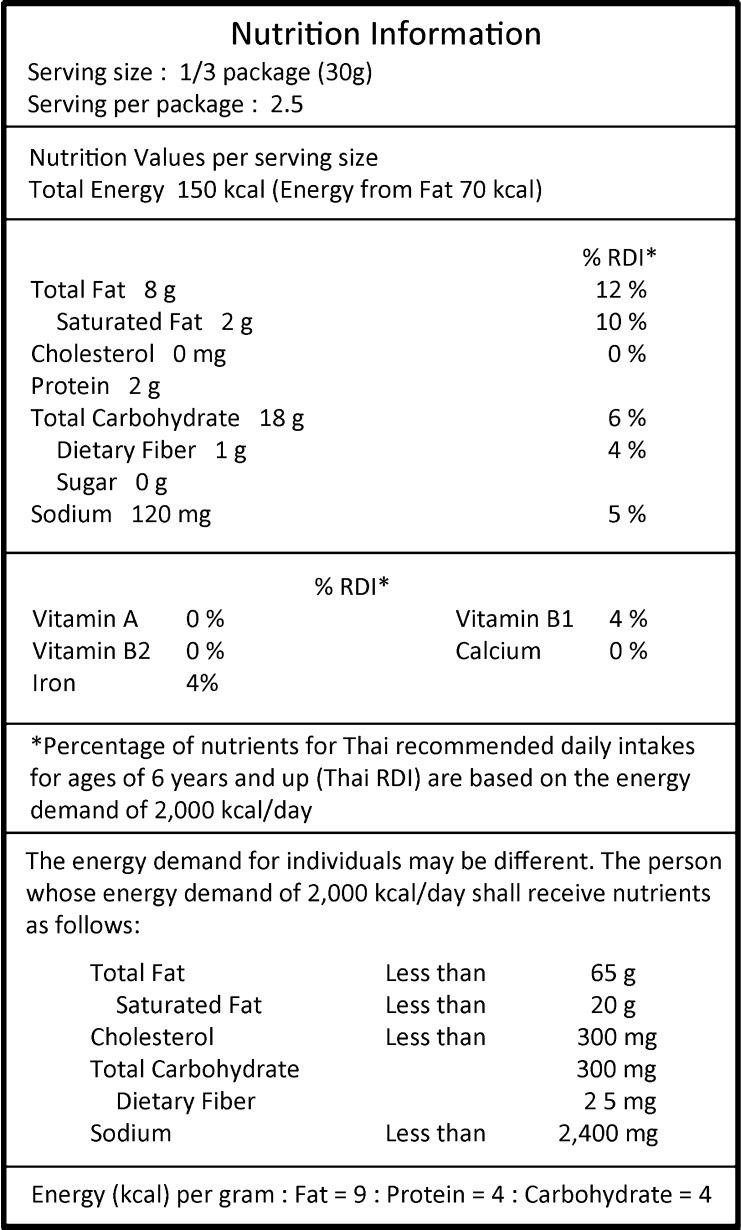
Nutrition Information Panel (NIP) translated from Thai label on a real snack.

As noted at the start of this section, Thailand first developed a Nutrition Information Panel (NIP) (Fig. 3) in 1998. In the beginning, foods making health or nutrition claims, foods for special diets, and foods using nutrition for marketing purposes were subjected to mandatory nutrition labels (Royal Thai Government Gazette, 1998). Thai nutrition labels have followed the Codex principles including declaration of nutrients, and nutrient calculation of the information (Codex Committee on Food Labelling, 2013). The appearance of the box in Thai nutrition labels was rather similar to USA nutrition labels. At that time, USA nutrition labels were leading for clarity and many countries were influenced to produce similar labels (Kongchuntuk, 1996). The full-format of a Thai nutrient data display box includes 15 items, but a short-form data display box can be used when some of the 15 nutrients are absent (Royal Thai Government Gazette, 1998).

Since 1998, the Thai government had paid great attention to nutrition labels increasing consumer’s knowledge. Nutrition labels appeared in mass circulation magazines and newspapers such as Thairath, Matichon, and Folkdoctor magazine as well as on television. Videos, brochures, and magnets were distributed to high schools in Thailand (Thai FDA, 2001). The book “10 steps to nutrition labels” was provided to food producers in 1999 (Kongchuntuk and Intaraluk, 1999). Some newspapers or magazines ran a quiz competition based on the information imparted and this created some enthusiasm in younger age groups. However, in 2007, INMU surveyed 1330 consumers and it showed that only 20% understood all the information on a NIP (Puwastien, 2008).

### Challenges for Thai nutrition labelling: traffic lights and GDAs

Thailand faces several unresolved issues regarding nutrition labelling. Over the last decade, many groups have advocated traffic light nutrition labels. Others oppose them because of lack of agreement on the “colour” of specific foods or on typical amounts ingested. Unfortunately, many less educated consumers also found NIPs were hard to comprehend. In 2006, the WTO Technical Barriers to Trade agreement (G/TBT/N/THA/215) was invoked to oppose a traffic light system in Thailand (Thai FDA, 2006; WTO, 2007). Investigating further, in 2007, Thai researchers explored a star system (1–5 stars), multiple traffic lights, and various nutrient models. This work was done with 450 Thai participants. The traffic light was favoured by participants as the most comprehensible and appropriate model (Sirichakwal, 2007).

**Fig. 4:**
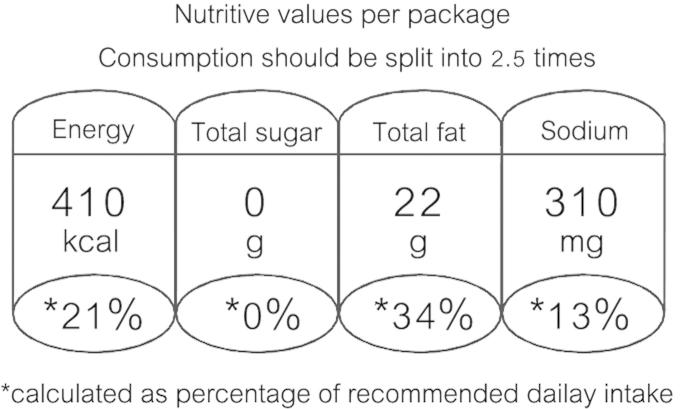
Guideline Daily Amounts (GDA) translated from Thai GDA label on a real snack.

Although many consumer organizations tried to push traffic light nutrition labels, they were abandoned as a nutrition information tool in Thailand. An additional Thai FDA study of other systems in 2009 showed Guideline Daily Amounts (GDA) (Fig. 4) was a good solution (Hokiarti, 2012).

However, GDA labels were not accepted by all. In 2010, the National Health Assembly (NHA) and the Thai cabinet agreed to manage the emerging problem of obesity in Thailand (Banwell et al., 2009). They proposed that the National Health Commission office (NHCO) and the Thai National Food Commission (TNFC) should support traffic light symbols on foods containing fat, sugar, or sodium (National Health Assembly, 2009). In 2011, eight Thai health organizations and many parents also signed a petition to the prime minister and the minister of MOPH asking for a traffic light label policy. But the Federation of Thai Industries argued that such traffic lights may induce consumers to eat too much “green foods” (Matichon newspaper online, 23 March 2011). Finally, the GDA label rather than the traffic light system was announced by a Thai FDA Notification of Labelling of Certain Ready-To-Eat Food (No. 2) in 2011 and it became effective on 24 August 2012 (Royal Thai Government Gazette, 2011).

Since 2011, GDA labels have been promoted by many campaigns to support consumer good eating and health. There have been GDA label brochures prepared by Thai FDA. As well, the GDA labels have been promoted by Thai FDA road shows at hypermarkets (Tesco Lotus) (Thairath newspaper (online), 21 April 2012) and the “Rai poong” project (Network of Fatless Belly Thais) (Chavasit et al., 2013). A Low Salt Thailand project is also underway (Supornsilaphachaii, 2013). As well knowledge is being transmitted through “Oryor noi” Youth FDA volunteer project (Hokiarti et al., 2012) and the “DekThaiDD” project developed by Nestle (Healthy Thai Kids project).

Because mandatory GDA labels may help consumers understand nutrition better, the Thai FDA designed these labels to cover all foods containing high sugar, fat, and salt. At first, five groups of snack foods were selected as pioneers for GDA labelling. There included fried or baked potato chips, fried or baked popcorn, rice crisps or extruded snack, crackers or biscuits, and filling wafers (Royal Thai Government Gazette, 2011). With good feedback from food producers, about 75% of targeted snack foods quickly developed GDA labels for their food packages within one year after the law became effective (Kumsri et al., 2013).

The Thai FDA conducted consumer surveys and published a summary of results at the 7th Thailand Congress of Nutrition, claiming that about 63% of those studies revealed that the participants had correctly understood information on the GDA labels (Yodtheun et al., 2013). Accordingly, the GDA policy remains in force for the five groups of snack foods and (in mid-2015) expansion to other foods is under consideration. It is expected to become mandatory for other food groups in Thailand including all snack foods (including peas and nuts, seaweed, and fish snacks), chocolate in all its forms, bakery products, semi-processed foods, and chilled and frozen ready-to-eat meals (Ministry of Public Health, 2013).

The Thai FDA plans to change labels soon. The changes include a lower value for the recommended daily sodium intake (from 2400 to 2000 mg). As well, information about food serving size will expand to include a greater variety of foods such as seaweed products. Furthermore, a trans-fat declaration will be included on future Thai nutrition labels (Parinyasiri, 2013). Thai FDA has also indicated plans to expand food labels by adding more about nutrient function claims (International Life Sciences Institute (ILSI) Southeast Asia Region, 2014).

### International tensions for food and nutrition labelling

#### Regional agreements and food trade

Food is an important component of global trade and has been a prominent part of trade agreements. Over the last three decades, regional trade agreements have appeared all over the world including North and South America, the Andean Community, the Caribbean, Eurasia, East Africa, and Asia. In South East Asia, ASEAN created a Free Trade Area (AFTA) over twenty years ago and aimed for economic integration by 2015. To achieve this, it will be necessary to agree on labels for traded food. But this complex work has not been completed partly because it involves agreement among countries ranging from low income to high income. The challenge confronting AFTA is an example of the food agreements that must be made in many other parts of the world as regional economic integration proceeds.

#### Harmonizing labels in Southeast Asia

Harmonizing food and nutrition labelling in Southeast Asia requires region-wide acceptance of international standards that are yet to be agreed. It will reduce trade barriers and consumer confusion but will involve tedious and prolonged negotiations. Many bureaucratic barriers persist among Southeast Asia countries including differences in scope of label regulation, variation of nutrient standards, and non-uniformity of nutrition labels. Issues surrounding labelling of processed foods have been discussed many times and are well documented by the recent report from the 8th Seminar on Nutrition Labelling, Claims and Communication Strategies (International Life Sciences Institute (ILSI) Southeast Asia Region, 2014).

Thailand has developed regulations over a long period and its rules regarding claims and Nutrition Information Panels are similar to Indonesia, the Philippines and Singapore. Vietnam has less experience and focuses regulations on milk product for children. Gradually, as each ASEAN country develops its own food and nutrition label standards, inter-country differences become apparent and constitute potential non-tariff barriers.

At present, each country has different nutrient reference values and rules regarding nutrition and health claims. For example, nutrient content claims for enrichment products are allowed in Malaysia (Gautier, 2010). Reference values involve Recommended Daily Intake (RDI) for Thailand, and Recommended Energy and Nutrient Intake (RENI) for the Philippines. Although Southeast Asia Recommended Dietary Allowances (SEA-RDAs) have been established, they are not yet integrated with Codex guidelines and not yet used for uniform labelling (Barba and Cabrera, 2008; Tee and Florentino, 2005).

All Southeast Asian countries follow Codex guidelines but have different ways of expressing nutrient content. Some nutrition labels list only a few nutrients, others show 15 nutrients or more (Tee et al., 2002). Labelling of core nutrients also differ. Energy, fat, protein and carbohydrates are the four core nutrients listed in Malaysia, the Philippines, and Singapore whereas Indonesia also lists sodium as the fifth core nutrient (Gautier, 2010). If food products are sold across Southeast Asian countries with source country nutrition labels consumers will be even more confused than they are dealing with labels produced by their own country.

#### National and international food label regulations

Complaints to WTO of Technical Barriers to Trade (TBT) show how labelling for consumer protection can be perceived as a trade barrier. Every country has a legal obligation to comply with WTO rules but this can conflict with sovereign responsibility and national public health laws (Albert, 2009). Frequent examples include conflicts related to nutrition labelling, ingredient list labelling, and country-of-origin labelling. Many TBT questions relate to definitions (e.g. organic foods), product categories (e.g. snack foods), and proof of claims (e.g. transgenic foods). This is well summarized in recent reports from the USA (United States Trade Representative, 2013, 2014).

The great variety of TBT complaints about food labelling regulations reveal the current tensions arising from food trade. Labelling that warns consumers about risks associated with the products were the source of frequent complaints. For example, Chile, Ecuador, and Peru tried to mandate front-of-package labelling for products with a high content of sugar, fat, or salt. The USA objected to such nutrition “stop sign” labels because they discourage consumption even if the product is not harmful when consumed in moderation (Grocery Manufacturers Association, 2014). Also, the European Union and Taiwan required labelling for transgenic foods but this led to TBT complaints due to a negative impact on trade that is not science based. When scientific evidence supporting labels is insufficient countries have revised them if they have the capacity to gather the necessary data. But in TBT complaints, developing countries are always disadvantaged due to lack of the necessary scientific expertise and resources (Anonymous, 2000). This disadvantage in obvious for TBT disputes involving South East Asia. Thailand can get entangled in first world-third world disputes as it has considerable expertise in food sciences and is also a major global food trader.

Concerns were often raised by many stakeholders whenever food regulation mandates labelling that reveals some attribute of food that would decrease consumption of the product. For example, Thailand was the first country to propose multiple traffic light nutrition labelling for snack foods in 2006. Some said it would cause confusion and mislead consumers. Finally, reflecting the interest of the food industry, Thailand later opted to implement a GDA system of labelling (Chavasit et al., 2013; Friel et al., 2013; Royal Thai Government Gazette, 2011; Thai FDA, 2006). In January 2013, Chile proposed a stop sign variant for foods “high in” fat, sugar, calories, or salt. Eleven countries including the USA raised concerns that the regulation was unclear, not scientific, and unnecessary to communicate the nutrient content of product. Chile continues to propose stop sign guided labels (Grocery Manufacturers Association, 2014; United States Trade Representative, 2014). After two years dispute, stop sign labels were signed into law by the Chilean President on April 2015 but WTO still considers the matter (Ramirez and Katial, 2015).

Beyond the Codex and the WTO, there are additional issues related to labels that can lead to disputes. For example, the USA is worried about the EU implementing “place of farming” labelling. The USA complained because there is no international guideline and it is difficult to comply for foods with multiple ingredients. Conversely, the USA also had country-of-origin labelling applied to imported beef and pork but these labels were rejected by WTO because of unfair adverse effects on imported meats from Canada and Mexico (Locke, 2015). Also, there have been complaints that Indian food labelling for “date of production” departs from the Codex Standard. And while Ecuador, Peru, Taiwan, and Russia are calling for “biotechnology labelling” others counter that biotech (transgenic) foods need not be treated differently (Codex Alimentarius, 2011). Warnings to “avoid excessive consumption” are criticized because they create fear in consumers (United States Trade Representative, 2014).

## Conclusion

Thai food labels have evolved for over 100 years to improve the quality and safety of food products. But the labels did not indicate nutrient content and daily requirements until the 1990s. These “nutrition labels” follow international trends to promote consumer health. Thai nutrition label policy follows the international Codex Alimentarius guidelines. A good impact on dietary behaviour, and eventually on health and nutrition, remains the elusive goal.

Thai food label regulation has changed in parallel with social change and economic development for the past century, while the country underwent a transition from a traditional subsistence agricultural society to a modern manufacturing middle income state. Within Thailand, sovereign power to draft national regulations for food and nutrition labels to protect consumers is now constrained by international trading policies and standards that have intruded. Strong consumer sentiment influenced food labelling 40 years ago but now international “fair trade” is a higher priority.

Managing different stakeholders has become a prominent issue. Thai food label policies now must find a balance among domestic consumer protection, nutrition promotion, abolition of international trading barriers, and adherence to international rules. These tasks also contribute to a national goal to make Thailand a kitchen to the world. This ambitious goal must be balanced by the ongoing commitment to optimize nutrition of the Thai population.

The evolution of the Thai food industry and rules that govern domestic and international trade have interacted with economic development in complex ways documented here and beginning a century ago. The experience of Thailand, which we could capture due to the good record systems, has useful information for many other countries, especially those with limited or confusing historical records. The Thai experience overlaps that of many other countries making similar transitions. Issues confronting the Thai food trade almost certainly affect food trade in other middle income countries with similarly strong traditions.
